# A Question of Smoke

**Published:** 1888-06-30

**Authors:** 


### THE DOCTOR'S PULPIT.

### A QUESTION OF SMOKE.

THE fascination of smoking is certainly an eccentric one. To draw into and then expel from one's mouth a sharp pungent substance seems about as strange a pleasure as the wildest imagination can dream of, and considering by what hard degrees it becomes a pleasure at all, it is rather to be wondered at that the fashion of smoking so persistently holds its own. Even if the successive steps that lead from the mild cigarette of the beginner to the deep-coloured meerschaum of the seasoned smoker can be traversed without a pang, or even the faintest giddiness, the satisfaction to be obtained from smoking is wholly indefinable. Yet undeniably the satisfaction is there, and the appetite for smoking can grow to such an extent, that some men would as soon go without their dinner as without their post prandial pipe.

Preachers and philosophers have inveighed against smoking till they are tired, and their audience too. They have explained all the mischief it might do to man's mind, body, and outward estate; man listens and smokes on. Sometimes the arguments employed are not of the most successful order. The present writer knew an old lady who assured her nephew, whom she had not seen between the ages of ten and twenty, that smoking would stunt his growth. He happened to be seated on a low chair, and perhaps seemed to her aged eyes to be rather a dwarfed specimen of humanity; but when he rose up with the remark, "Do you think so, aunt?" and stretched himself out to his six-feet-two of broad-shouldered manhood, she was silenced, if not convinced. The argument that the use of tobacco shortens life has been successfully used, though perhaps a certain anecdote bearing on that point may be too good to be true. "You say that tobacco shortens life?" said a veteran smoker to the leader of an anti-tobacco crusade. "I have smoked for fifty years, and I have managed to live till I'm seventy." "Ah! if you hadn't smoked you might have been eighty by this time," is the answer the unlucky crusader is reported to have made. In spite, however, of this old gentleman's good luck, it must be conceded that for the young, at least, smoking is a very dangerous, pernicious habit, that often permanently injures the health.

Tobacco is, however, a thing regarding which it is impossible to lay down any absolute law. It is technically a narcotic poison, and its active principle, nicotine, is undeniably deadly in its action. But in practice it is for most people such a very slow poison that a moderate smoker is in little more danger of dying from nicotine poisoning than he would be of dying from the action of prussic acid, because he partook of blanc-mange flavoured, after the fashion of our grandmothers, with bay leaves. To some constitutions indeed tobacco is an active poison—bad for the heart, bad for the digestion, bad for the sight, bad for the nerves; but these are few, and if their owners have not the wisdom and self-control to give up the habit of smoking when they find it injuring them their weakness may demand our pity, but cannot convince us that because they suffer, the sociable pipe—the calumet of peace among civilised as well as savage races—should be banished for ever from the land. The doctor has not a word of reproach to cast at the moderate smoker, and will even give him the comforting assurance that his after-dinner cigar will, by increasing the secretion of saliva, absolutely aid digestion. He must add, however, that if the said saliva be recklessly wasted by the unnecessary and uncleanly habit of spitting, smoking will retard digestion, the food being sent down to the stomach without being sufficiciently mixed with saliva, and the gastric juice thereby over-taxed.

But the inveterate smoker, the man who lives only to smoke, the man who exists only as an appendage to his pipe—he is another sort of individual altogether. He must be reminded that there is no habit, however innocuous when kept under due restraint, which does not become mischievous when indulged in to excess. When a man gets into the condition of thinking that every place where he cannot enjoy the active companionship of his pipe or cigar, every person in whose presence he may not smoke, is a blot on the face of creation—and it is to be feared that such slaves to the habit are known to us all—he is on the high road to suffering from nicotine poisoning—and serve him right. He must be told that the volatile oil whose pungency tickles his palate, will paralyze his nervous system, while the nicotine, which makes him protest so loudly when by an unlucky chance he tastes a few drops of it, will injure his heart and brain. Whether it be in food, drink, work, pleasure, or anything else, the man who forgets the due proportion of things must in the long run pay the penalty of his indulgence. "There is nothing more terrible than outraged nature," says Charles Kingsley—nature, patient and long-suffering, but inexorable at the last.

But, as was said before, the denunciation of excess does not imply the condemnation of moderate indulgence, and smoking has a good many things to be said in its favour. It has the useful quality of staying the pangs of hunger in those accustomed to its use. In the Franco-German war the German soldiers managed, by the help of tobacco, to do without food for hours while executing long marches and undergoing great fatigue. The second battle of Orleans was, it is said, fought and won on tobacco, as the transport department had broken down, and the German army was without proper rations for thirty-six hours. It lures many a busy man into taking half an hour of necessary idleness. The cynic who described fishing as "the waste of a good opportunity for doing nothing," might probably say the same of smoking; but, in fact, it is an improved edition of doing nothing; it gives the necessary cessation from work without the remorseful, conscience-pricking feeling of being lazy. And smoke is the sign and seal of companionable silence. Friends, host and guest, may pass happy hours together thus, the former not fatigued by the effort of entertaining, the latter not bored by the lack of it, all needful sociability expressed by the occasional handing of a match or the exchange of a tobacco-pouch. How restful is the companionship that does not demand conversation need not be said. It is peace without loneliness, silence without solitude. Is it not recorded how Carlyle and Tennyson once spent hours together without uttering a word, content with exchanging the discourse of puffs and smoke-wreaths? At last the poet rose to go. Then the philosopher, the preacher of the gospel of silence (in thirty volumes) found a voice. "Eh, Alfred, man," he exclaimed, "we've had a grand evening; come again soon." And who shall say that the thoughts bred under the sweet influence of nicotine in the minds of such men, or even men of lesser calibre, are, in the end—only smoke.

